# Current–Phase Relation of a WTe_2_ Josephson Junction

**DOI:** 10.1021/acs.nanolett.3c01416

**Published:** 2023-05-08

**Authors:** Martin Endres, Artem Kononov, Hasitha Suriya Arachchige, Jiaqiang Yan, David Mandrus, Kenji Watanabe, Takashi Taniguchi, Christian Schönenberger

**Affiliations:** † Department of Physics, University of Basel, Klingelbergstrasse 82, 4056 Basel, Switzerland; ‡ Department of Physics and Astronomy, University of Tennessee, Knoxville, Tennessee 37996, United States; § Material Science and Technology Division, Oak Ridge Laboratory, Oak Ridge, Tennessee 37831, United States; ∥ Department of Materials Science and Engineering, University of Tennessee, Knoxville, Tennessee 37996, United States; ⊥ Research Center for Functional Materials, National Institute for Materials Science, 1-1 Namiki, Tsukuba 305-0044, Japan; # International Center for Materials Nanoarchitectonics, National Institute for Materials Science, 1-1 Namiki, Tsukuba 305-0044, Japan; ∇ Swiss Nanoscience Institute, University of Basel, Klingelbergstrasse 82, 4056 Basel, Switzerland

**Keywords:** WTe_2_, topological superconductivity, higher-order topological insulators, edge states, current−phase relation, asymmetric SQUID

## Abstract

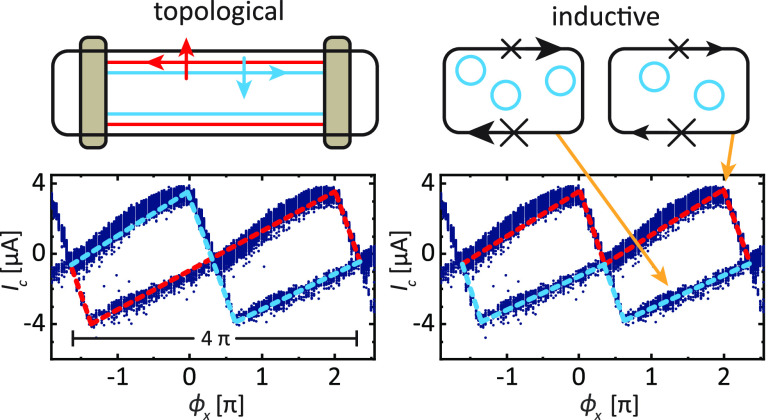

When a topological insulator is incorporated into a Josephson junction, the system is predicted to reveal the fractional Josephson effect with a 4π-periodic current–phase relation. Here, we report the measurement of a 4π-periodic switching current through an asymmetric SQUID, formed by the higher-order topological insulator WTe_2_. Contrary to the established opinion, we show that a high asymmetry in critical current and negligible loop inductance are not sufficient by themselves to reliably measure the current–phase relation. Instead, we find that our measurement is heavily influenced by additional inductances originating from the self-formed PdTe_*x*_ inside the junction. We therefore develop a method to numerically recover the current–phase relation of the system and find the 1.5 μm long junction to be best described in the short ballistic limit. Our results highlight the complexity of subtle inductance effects that can give rise to misleading topological signatures in transport measurements.

Topological insulators (TIs) belong to a unique class of materials that are insulating in their bulk while hosting gapless boundary states that are protected by time-reversal symmetry.^1^ The class of three-dimensional TIs has recently been extended,^2,3^ realizing that a *d*-dimensional TI of order *n* can develop (*d* – *n*)-dimensional hinge or corner states. A promising candidate of this novel material class that is predicted to host topological hinge states is the semimetallic transition-metal dichalcogenide WTe_2_.^4−7^ While bulk states dominate transport in the normal state, hinge states become the governing transport channel over long distances in the superconducting state, as they can carry a higher critical current due to their reduced dimensionality.^8,9^ Therefore, Josephson junctions (JJs) formed by a TI as the weak link between two superconducting electrodes provide an ideal platform to probe the topological nature in a transport experiment. Topological hybrid systems are of great interest, as they are predicted to host unconventional superconductivity,^10^ the fundamental building block of a potential topologically protected quantum bit.^11^ The fingerprint of a JJ is the current–phase relation (CPR), the dependence of the supercurrent on the phase difference φ between the superconducting electrodes. The measurement of the CPR directly reflects the underlying transport mechanism with which the Cooper pairs are shuttled across the weak link. For a topological weak link, perfect Andreev reflection is expected,^12^ since spin-momentum locking in the hinge states prohibits normal electron reflection at the interface to the superconductor. In the long junction limit, the supercurrent is carried by 4π-periodic Andreev bound states of opposite parity, σ^+^ and σ^–^, that give rise to a characteristic sawtooth-shaped *I*_c_. Parity conservation prohibits the recombination to the lower energy branch and results in a multivalued *I*_c_ with a distinct diamond shape, as plotted in Figure 1a.^13^ For comparison, the expected CPR of a trivial ballistic JJ in the long junction limit is plotted as a black dashed line in the same figure. Accordingly, the literature often infers ballistic topological states from the observation of a sawtooth-shaped flux dependence of the critical current.^9,14−16^

**Figure 1:**
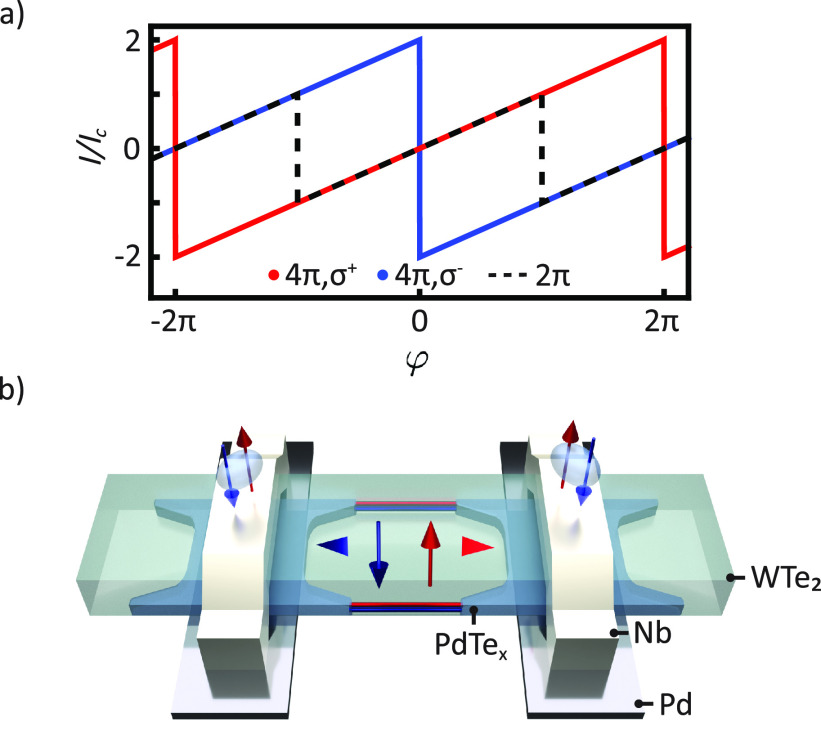
CPR of a topological JJ. (a) Normalized CPR of a topological JJ in the long junction limit as a function of the junction phase φ. The 4π-periodic supercurrent through the SQUID is carried by Andreev bound states of opposite parity, σ^+^ and σ^–^, resulting in a multivalued switching current that resembles a diamond-like pattern. A 2π-periodic CPR of a topologically trivial junction is shown as a dashed line for comparison. (b) llustration of a JJ fabricated from an elongated WTe_2_ crystal on top of Pd bottom contacts. Superconducting Nb contacts were deposited from above after etching through the top hBN (not displayed). The diffusion of Pd into the WTe_2_ crystal leads to the formation of superconducting PdTe_*x*_ inside the weak link, through which the topological hinge states can be coupled.

Recently, high-quality JJs formed in WTe_2_ on palladium (Pd) bottom contacts have been reported^17,18^ and provided evidence of a nonsinusoidal CPR.^7,19^ Here, we combine such JJs based on Pd-induced superconductivity in WTe_2_ with external superconducting leads, as illustrated in Figure 1b. A superconducting quantum interference device (SQUID) formed out of two such JJs is expected to reflect the CPR, provided that the critical current amplitudes of the two JJs are highly different and the loop inductance is negligible.^20,21^ Based on this method, we observe a switching current distribution with 4π-periodicity that resembles a topological JJ.^13^ Contrary to the topological interpretation, we provide an alternative explanation based on inductance effects,^22^ which could be relevant for a number of previous experiments.^9,14−16^ Importantly, the inductance contribution originates from the JJs themselves and exceeds the loop inductance. We further developed a numerical model based on the maximization of the supercurrent in the SQUID loop that allows us to exclude screening effects from the additional inductances and recover the real CPR. The calculations suggest that the critical current of our 1.5 μm long junction is best reproduced in the short ballistic limit, despite its long physical length.

We begin with the fabrication of an asymmetric SQUID out of WTe_2_. In our experiment, both JJs are formed in the same needle-shaped WTe_2_ flake of width *w* = 1.5 μm. The asymmetry in critical current *I*_c_ of the two involved JJs is achieved by a different spacing between the Pd bottom contacts *l*_w_ = 1.5 μm and *l*_r_ = 0.5 μm for the weak and reference junction, respectively, as sketched in Figure 2a. The superconducting loop is formed by etching through the top hBN into WTe_2_ and sputtering niobium (Nb) on top. The Nb leads are between 2.2 and 3 μm wide and 100 nm thick. A detailed description of the fabrication process can be found in the Supporting Information. Figure 2a displays an optical image of the finished devices. In the following we will focus on the lower SQUID, enclosed by the dashed line.

**Figure 2:**
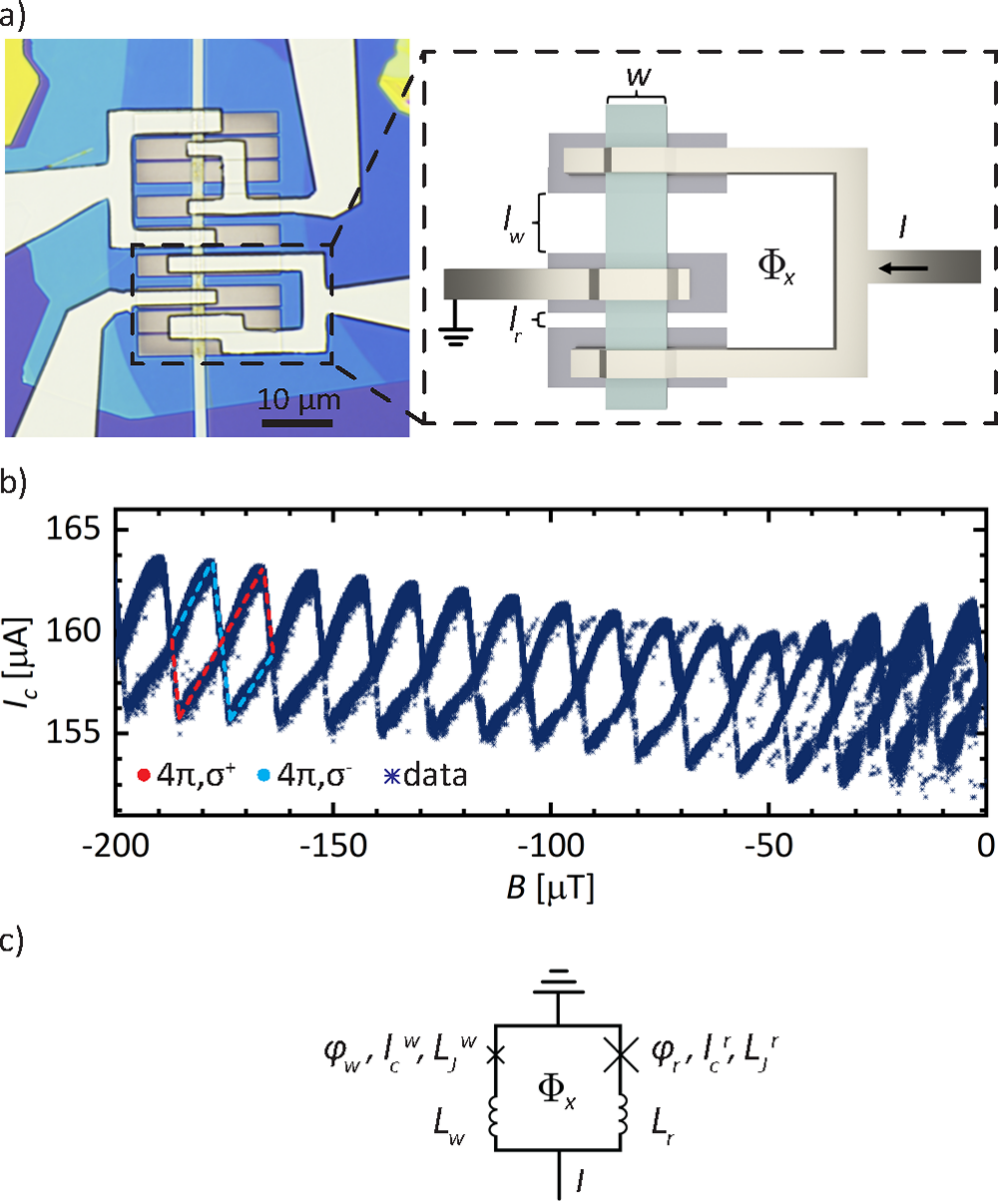
Switching statistics of the weak junction in an asymmetric SQUID. (a) Optical image of a SQUID device on the left, with the measured SQUID highlighted by the dashed box. An illustration of the device parameters is provided to the right. (b) High-resolution measurement of the SQUID switching current as a function of applied magnetic field through the loop. Oscillation periods of the parity states σ^+^ and σ^–^, respectively, are highlighted in red and light blue, respectively. The period δ*B* = 23.2 μT of a single parity branch displays a 4π-periodicity with respect to the designed loop area δ*B* = 11.1 μT. (c) Schematic of the SQUID, specifying the device parameters, including the additional series inductances *L*_w_ and *L*_r_ in series to the weak and reference JJ, respectively.

The critical current of the SQUID is given by the sum  of the individual currents *I*_w_ and *I*_r_ through the two branches of the loop, defined by the critical current *I*_c_^i^ and the normalized CPR *f*_i_ of the *i*th Josephson junction. The total flux Φ_tot_ threading the loop connects the phase differences across the two JJs φ_w_ – φ_r_ = 2πΦ_tot_/Φ_0_ = ϕ_tot_, with ϕ_tot_ denoting the external phase. In the absence of inductances in the loop, ϕ_tot_ is simply defined by the external phase ϕ_*x*_. The asymmetry *I*_c_^r^ ≫ *I*_c_^w^ pins φ_r_ = φ_r_^max^ at a fixed phase, for which *I*_c_^r^ is maximized.^20,21,23^ The normalized CPR of the weak junction *f*_w_ can then be deduced from the measurement of
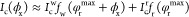
1In the experiment, Φ_*x*_ = *BA*_o_ is controlled by an applied perpendicular magnetic field *B* threading the effective loop area *A*_o_ = 186 μm^2^. The Meissner screening of the enclosing superconducting loop was taken into account by including half of its width into the loop area. The final device is probed in a quasi four-terminal configuration by sourcing an ac current and monitoring the voltage drop over the SQUID. We use the counter technique, as described in the Supporting Information, to measure the switching statistics of the device.

Figure 2b presents the measured switching statistics of the SQUID in an extended magnetic field range at base temperature *T* = 30 mK of the cryostat. Visible is a multivalued *I*_c_ that oscillates periodically around the offset current *I*_c_^r^ = 160 μA of the reference junction. Given the great difference in critical current amplitudes *I*_c_^r^/*I*_c_^w^ ≈ 40, we expect the SQUID to be highly asymmetric and the measured signal to reflect the CPR of the weak JJ. A second set of oscillations appears for field values from *B* = −100 μT upward and is explained in the Supporting Information by the behavior of the reference junction. The extracted oscillation period of a fixed parity branch, as defined in Figure 1a, is equal to δ*B* = 23.2 μT and therefore twice the value δ*B* = Φ_0_/*A*_o_ = 11.1 μT expected for the enclosed loop area *A*_o_. The data represent striking resemblance to the two parity states of a topological JJ in the long junction limit with a 4π-periodicity in flux. However, the amplitude of the signal deems this explanation unlikely. A single channel in the topological ballistic long junction can carry a current *I*_c,4π_ = *E*_Th_*e*/*ℏ* = *v*_F_*e*/*l*,^13^ with *E*_Th_ being the Thouless energy, *v*_F_ the Fermi velocity, and *l* the length of the weak link. *I*_c_ in our junction would therefore have to be carried by at least  116 perfectly ballistic channels in parallel, assuming *v*_F_ = 3.09 × 10^5^ m s^–1^.^24^ WTe_2_ is expected to host a pair of hinge states on opposite edges of the crystal.^19^ Conducting states have been found to reside at step edges in the crystal where the number of vdW layers changes,^7^ but we do not observe such crystal steps in optical microscopy for the used flake. We conclude that it is unlikely for the current to be carried purely by ballistic hinge states.

Instead, multivalued CPR measurements have previously been reported in devices containing a superconducting weak link with large kinetic inductance that is responsible for strong screening effects.^22,25−28^ In strong contrast to previous experiments, however, the device studied here contains a normal conducting weak link^18^ and has negligible loop inductance, as will be shown later.

The schematic in Figure 2c introduces a set of additional inductances, *L*_r_ and *L*_w_, that are placed in series to the reference and weak junction, respectively, while potential mutual inductances are assumed to be negligible. In general, the total phase
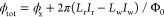
2can differ strongly from the phase created by the external flux ϕ_*x*_ = 2πΦ_*x*_/Φ_0_, due to the contribution induced by the currents *I*_r_ and *I*_w_ passing through the inductances in the SQUID arms, *L*_r_ and *L*_w_. We note that while a screening current can distort the flux dependence of the critical current, it does not change its periodicity.^29^

Deducing the CPR from the inductive SQUID measurement requires the knowledge of the phase dependences of *I*_r_(ϕ_tot_) and *I*_w_(ϕ_tot_) themselves. In order to bypass this recursive constraint, we make an assumption about the CPRs of the JJs that is based on the experimental data. We are then going to use this information in the next step to calculate , for which *I*_c_(ϕ_tot_) is maximized according to eq 1. Last, we are going to include screening effects in the model and obtain the relation *I*_c_(ϕ_*x*_), which is placed in context to the experimental data.

Our choice of CPR for the weak junction is based on two experimental observations. First, the rising slope of *I*_c_(ϕ_x_) is nonlinear, suggesting the same should hold for the CPR. Second, *I*_c_(ϕ_x_) has self-crossings, implying that the phase of the reference junction does not remain fixed, contrary to the established expectation for a highly asymmetric SQUID. The behavior is possible if the CPR of the weak junction contains abrupt changes, such as is the case in the ballistic limit of the CPR. We illustrate this behavior later in the text and provide an additional discussion in the Supporting Information. *I*_w_(φ_w_) is modeled to be in the 2π-periodic, ballistic short junction limit^30^
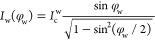
3scaled by the amplitude of *I*_c_^w^. The exact CPR of the reference junction plays little role in the further discussion, yet, since it is formed in the same material but with reduced length, we also model it as a short ballistic junction, scaled by *I*_c_^r^. Independently, we have used length-dependent measurements in the Supporting Information and the analysis of the PdTe_*x*_ diffusion profile in ref (18) to verify that both junctions behave indeed as JJs and are not shorted by PdTe_*x*_.^31^

Having made this initial assumption, we continue to calculate the resulting *I*_c_ by maximizing the current through the SQUID. The top panel in Figure 3b illustrates a visual method to maximize the critical current as a function of ϕ_tot_. The individual currents through the SQUID arms *I*_r_ and *I*_w_ follow eq 3. The currents evolve in opposite flux direction, due to the connection φ_r_ – φ_w_ = ϕ_tot_. We start at the configuration ϕ_r_ = ϕ_r_^max^ and ϕ_w_ = ϕ_w_^max^, when the currents through the reference and weak junctions are at their maximum, *I*_c_^r^ and *I*_c_^w^, respectively. Moving from this point in the negative direction of ϕ_tot_, *I*_c_ follows *I*_w_, plotted as a red curve. In the opposite direction toward positive values of ϕ_tot_, *I*_w_ faces a sudden drop. Instead of following *I*_w_, a higher *I*_c_ is obtained by following *I*_r_, drawn in dark blue, until the point when *I*_r_ intersects with *I*_w_. This creates a small flux range δφ_tot_ ∝ π*I*_c_^w^/*I*_c_^r^ ≪ π, in which *I*_c_ is maximized by following *I*_r_ rather than *I*_w_, resulting in a changing φ_r_, while ϕ_w_^max^ = π remains fixed. The situation is illustrated in the lower panel of Figure 3b. Once the two current branches cross with evolving flux, φ_r_ returns to its maximum value ϕ_r_^max^ = π and *I*_c_(ϕ_tot_) follows the flux dependence of *I*_w_. The well-known behavior of the asymmetric SQUID is restored. While δϕ_tot_ remains small in the above scenario, it can extend significantly in the experiment, due to the inductance effects, as described by eq 2.

**Figure 3:**
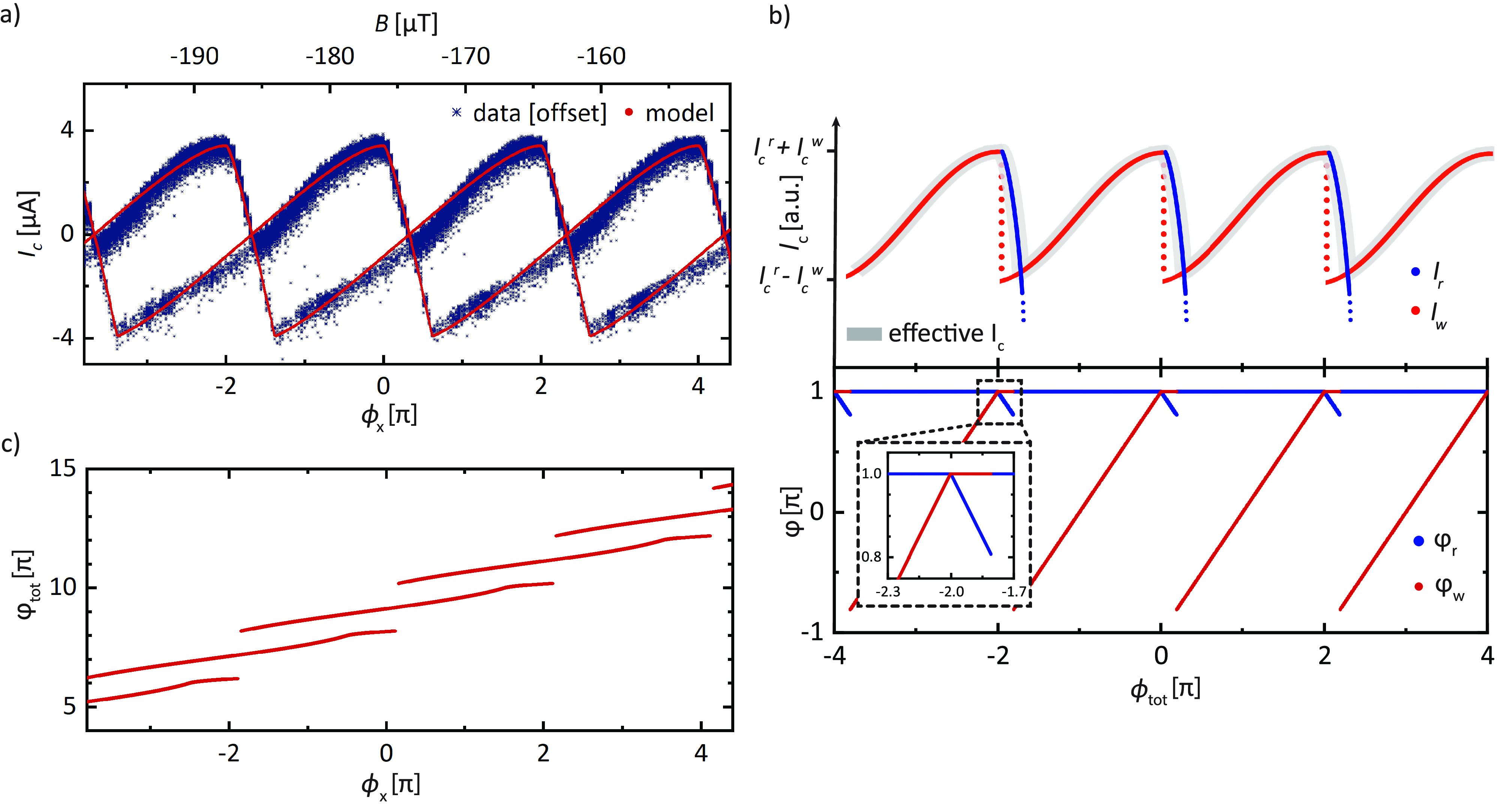
SQUID oscillations and numerical model of the CPR. (a) Experimental data of the SQUID oscillations as a function of external flux ϕ_x_ shown in blue. The red data points are a fit to the data based on maximizing the critical current in the SQUID loop. Phase winding in the superconducting loop is responsible for the multivalued supercurrent. (b) Visual method to maximize *I*_c_ as a function of total flux ϕ_tot_. The upper panel plots the currents *I*_r_ and *I*_w_ for a short ballistic CPR with an amplitude ratio *I*_c_^r^/*I*_c_^w^ = 40. The two currents evolve in opposite directions with increasing ϕ_tot_, due to φ_r_ – φ_w_ = ϕ_tot_. The resulting maximized *I*_c_ is highlighted by a gray background and is composed of the weak junction and the reference junction CPR for the rising and falling sides, respectively. The lower panel shows the corresponding behavior of the junction phases φ_r_ and φ_w_, obtained through the numerical maximization model. The phases are mapped to the range of the CPR. The switch between the observed weak and reference junction branch in the top panel is accompanied by a shift from a fixed φ_r_ to φ_w_ at flux values of multiples of 2π. (c) Numerically calculated ϕ_tot_ versus ϕ_x_ using the model described in the main text. Inductance effects give rise to the multivalued ϕ_x_(ϕ_tot_), responsible for the intertwined branches visible in (a).

Given that φ_r_ does not necessarily remain fixed in an asymmetric SQUID, we introduce next a numerical procedure to transfer the above model from the dependence of total flux ϕ_tot_ to the external flux ϕ_x_, the quantity applied in the experiment. The slope of *I*_c_(ϕ_x_) is directly related to the inductance of the current carrying arm in the SQUID, via , with *L*_J_^i^ being the Josephson inductance and i = r, w, depending on the considered data mapping the CPR of the reference or the weak junction.

First, we numerically maximize the expression
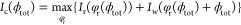
4with respect to φ_r_(ϕ_tot_) for a given ϕ_tot_. *I*_r_ and *I*_w_ correspond to the currents through the respective SQUID arms, i.e.  and . The two amplitudes *I*_c_^r^ = 160 μA and *I*_c_^w^ = 3.75 μA are determined from the fixed current background and the oscillation amplitude of the intertwined branches. The lower panel in Figure 3b plots the obtained φ_r_ and φ_w_ in blue and red, respectively. Based on the choice of the CPR function in eq 3, φ_r_ is mostly fixed at the maximum value ϕ_r_^max^ = π, while φ_w_ evolves linearly in ϕ_tot_, according to ϕ_w_ = φ_tot_ + ϕ_r_^max^. However, a small range δϕ_tot_ exists, where φ_r_ changes in flux while φ_w_ remains fixed, in agreement with the graphical method introduce above in the upper panel of Figure 3b.

Using φ_r_(ϕ_tot_), it is now possible to extract the inductance effects and recalculate ϕ_*x*_ = ϕ_tot_ – 2π(*L*_r_*I*_r_ – *L*_w_*I*_w_)/Φ_0_. Depending on the magnitude of the incorporated inductances and critical currents, self-inductance effects in the loop cause the connection ϕ_tot_(ϕ_x_) to become multivalued, as is visible from Figure 3c.

Finally, *I*_c_(ϕ_x_) is plotted in red in Figure 3a. Despite the long physical length *L*_w_ = 1.5 μm of the junction, we find the bending of the gradually rising slope d*I*_c_/dϕ_x_ to be well reproduced by *f*_w_ being in the 2π-periodic, ballistic short junction limit. In both cases, the magnetic field dependences of the current amplitudes are assumed to be negligible for the given field range. Importantly, despite the great difference in critical current amplitudes of the embedded junctions, the model confirms that φ_r_ does not remain fixed in flux. The experimental CPR of the SQUID is composed of the weak and the reference junction CPR. Even though the CPR of the weak junction is not necessarily uniquely in the short junction limit, it has to have a sharp transition in flux and therefore be close to ballistic in order to ease the shift between the fixed φ_r_ and φ_w_.

Further, we extract the inductances *L*_r_ = 60 pH and *L*_w_ = 220 pH, by matching the rising and falling slopes of the fit to the data. An important result that distinguishes ours from previous experiments is that *L*_r_ and *L*_w_ by themselves exceed the sum of geometrical inductance *L*_geo_ ≈ 27.0 pH^32^ and kinetic inductance *L*_kin_ ≈ 5.5 pH^33^ for the Nb SQUID loop. Possibly, additional JJs can form at the interface between the sputtered superconducting leads and the self-formed superconducting PdTe_*x*_,^34^ yet given that their critical current has to be larger than *I*_c_^r^, little inductance contribution is to be expected. Instead, we attribute the origin of additional inductance and its asymmetry between the SQUID arms to the superconducting PdTe_*x*_ that has self-formed at the interface between WTe_2_ and Pd. Further support of this interpretation is provided in the Supporting Information, including the comparison of the data to different initial CPR assumptions.

Finally, the multivalued *I*_c_ can also be explained in the framework of excited vorticity states in an inductive SQUID.^25−28,35^ Using the parameters obtained from our fit, we calculate the magnetic screening factor  1,^29^ reflecting that an additional flux quantum can be created by the maximum circulating current through the weak JJ. The result is a multivalued ϕ_tot_ as a function of ϕ_x_, as was shown in Figure 3c for the given device parameters. The above behavior can differ strongly even on a single sample chip. While the second SQUID loop formed on the same WTe_2_ flake (compare Figure 2a) reveals the same behavior of the reference junction with multiple branches, we do not observe higher-vorticity states in the SQUID oscillations. The absence of the feature is most likely connected to the overall smaller *I*_c_^w^, despite the shorter junction length with *l*_w_ = 1.2 μm.

In conclusion, the established assumption of a fixed reference junction phase in flux does not hold for highly transparent junctions, even in the case of highly asymmetric critical current amplitudes. Furthermore, we have shown the complexity of subtle inductance effects that reach beyond the standard consideration of the loop inductance and might create misleading topological features. It is therefore crucial for a correct CPR measurement to consider potential inductance contributions from the interfaces between the embedded junctions with the SQUID loop. The origin of such additional inductances can go beyond the diffusion of PdTe_*x*_ presented here and may include defects implanted at the interface through various fabrication steps.^33,36^ Despite these limitations, the fitting routine presented here allows reproduction of the experimental data closely. The best result was obtained by placing the weak junction in the short ballistic limit, as presented in the Supporting Information. Our results establish WTe_2_ as a promising platform for further experiments toward topological superconductivity.

## Data Availability

All data in this publication are available in numerical form in the Zenodo repository.^37^

## Supporting Information Available

Materials and methods, counter measurement technique, inductance effects in single Josephson junctions, verification of the inductance effects, comparison of the CPR configurations in the fitting procedure, instability of the reference junction phase φ_r_, and frequency dependence of the switching statistics (PDF)
