# Supplementary material for: Current–Phase Relation of a WTe2 Josephson Junction
Source: Nano Lett. 2023 May 8;23(10):4654–9. doi: 10.1021/acs.nanolett.3c01416 (PMC10214438; doi:10.1021/acs.nanolett.3c01416)
Supplement: Supplementary file 1 — nl3c01416_si_001.pdf [file nl3c01416_si_001.pdf]

Supplementary Information: Current-phase relation of a WTe_2 Josephson junction

Martin Endres,[1] Artem Kononov,[1] Hasitha Suriya Arachchige,[2] Jiaqiang Yan,[2,3] David Mandrus,[4,2,3] Kenji Watanabe,[5] Takashi Tanigutchi,[6] and Christian Schönenberger[1,7]

[1]*Department of Physics, University of Basel, Klingelbergstrasse 82, 4056 Basel, Switzerland*

[2]*Department of Physics and Astronomy, University of Tennessee, Knoxville, Tennessee 37996, USA*

[3]*Material Science and Technology Division, Oak Ridge Laboratory,Oak Ridge, Tennessee 37831, USA*

[4]*Department of Materials Science and Engineering, University of Tennessee, Knoxville, Tennessee 37996, USA*

[5]*Research Center for Functional Materials, National Institute for Materials Science, 1-1 Namiki, Tsukuba 305-0044, Japan*

[6]*International Center for Materials Nanoarchitectonics, National Institute for Materials Science, 1-1 Namiki, Tsukuba 305-0044, Japan*

[7]*Swiss Nanoscience Institute, University of Basel, Klingelbergstrasse 82, 4056 Basel, Switzerland*

(Dated: April 25, 2023)

I. MATERIALS AND METHODS

Stacking of the vdW Heterostructure

The following section describes the fabrication process of the devices, valid in general for both, singe JJs and SQUID loops. First, the palladium (Pd) bottom contacts are fabricated on a p-doped silicon wafer with 285 nm SiO_2 layer on top. This type of wafer is used throughout the complete fabrication process and is referred to as Si/SiO_2 wafer. The structure is written via e-beam lithography, followed by metal deposition in an e-beam evaporator of 3 nm titanium (Ti) and 15 nm Pd. The lift-off of excess metal is done in warm acetone at 50 °C for 1 hour. Next, the van-der-Waals materials are prepared. Hexagonal boron nitride (hBN) is exfoliated in ambient conditions on untreated Si/SiO_2 wafers using adhesive tape. Suitable flakes with a thickness between 10 nm and 30 nm are selected via optical contrast under the microscope [1] and are checked to be uniform in dark-field mode. WTe_2 is an air sensitive van der Waals material prone to oxidation [2–4]. Starting from exfoliation until complete encapsulation, the crystals are handled inside a nitrogen filled glovebox with an oxygen level < 1 ppm. We use flux grown, needle shaped WTe_2 crystals for exfoliation that are preferably oriented along their crystallographic a-axis [5]. The crystals are exfoliated using first an adhesive tape which is then pressed onto a polydimethylsiloxane (PDMS) stamp. The PDMS stamp is placed on a Si/SiO_2 substrate and the package is heated for 5 minutes at 120 °C on a hotplate. After cool-down, PDMS is peeled off from the substrate and fitting flakes with thickness between 10 nm and 30 nm are selected using their optical contrast and dark field imaging. We use a dry transfer technique [6] with a polycarbonate (PC)/PDMS stamp to successively pick up the selected hBN and WTe_2 flakes at $T = 80\,°C$ and release them on the patterned Pd bottom contacts by melting the PC at $T = 150\,°C$. Remaining PC residues are dissolved at room temperature for 1 hour in dichloromethane.

Deposition of Superconducting Contacts

Neither WTe_2 nor Pd are intrinsically superconducting and superconductivity only emerges when both materials are brought in contact to each other [7, 8]. The phenomenon arises through the diffusion of Pd into the WTe_2 crystal and the formation of superconducting $PdTe_x$ [7]. The external superconducting loops are fabricated using a recently developed recipe for high transparency junctions, as described in Ref. [7].

First, an etch mask is written by e-beam lithography, defining the later SQUID loop (or the contacts for single JJs) on top of the vdW stack. In order to preserve the high contact transparency [7], the superconducting contacts are separated by a distance $l_{\mathrm{Pd}} \approx 0.5\,\mu\mathrm{m}$ from the JJ interface defined by the Pd bottom contact. We use a CHF_3/O_2 plasma in a reactive ion etcher (RIE) to etch through the encapsulating hBN layer into the WTe_2. After the quick transfer to the sputtering machine, an argon (Ar) plasma at 50 W is ignited for 1 minute. Afterwards, 100 nm Nb with a capping layer of 3 nm platinum (Pt) capping layer are sputtered. Alternatively, 100 nm aluminium is deposited in an e-beam evaporator (also including a plasma etching step before).

II. COUNTER MEASUREMENT TECHNIQUE

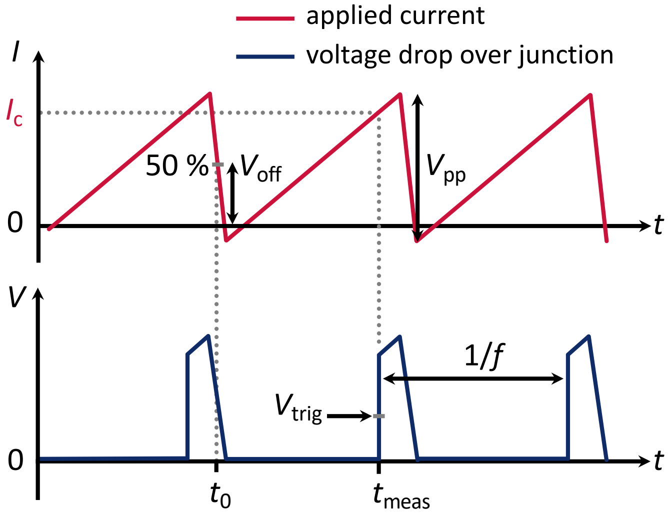

FIG. S1. **Counter measurement technique.** A sawtooth-shaped voltage V_{bias} is applied to a resistor R_{bias} in series with the device, effectively creating an alternating current I (upper panel). Simultaneously, the voltage V_{SQUID} (lower panel) over the device is measured and a non-zero value is detected when $I > I_{\mathrm{c}}$, the critical current of the SQUID. From the known input parameters of the ac-current it is possible to calculate the switching current.

The devices are probed in a quasi four-terminal configuration by sourcing an ac-current and monitoring the voltage drop over the SQUID. We use the counter technique, outlined in Fig. S1, to measure I_{s} and map the switching statistics of the device [9]. The sawtooth current I is created by applying a sawtooth-shaped voltage V_{bias} with frequency f (5 Hz –

1.7 kHz), amplitude V_{pp} and off-set voltage V_{off} from zero (upper panel) to a resistor $R_{\mathrm{bias}} \sim 10\,\mathrm{k}\Omega$ in series with the device. The voltage drop over the SQUID V_{SQUID} (lower panel) is measured and forwarded to a counter. For $I < I_{\mathrm{c}}$, the device resides in a dissipationless state and V_{SQUID} equals zero. Once $I = I_{\mathrm{c}}$ is exceeded, transition to the resistive state sets in sharply and a voltage is detected. I_{c} is calculated through

$$I_{\mathrm{c}} = \frac{1}{R_{\mathrm{bias}}}\left(V_{\mathrm{off}} + \frac{V_{\mathrm{pp}} f}{\mathrm{x}}(t_{\mathrm{meas}} - \frac{1}{2f}\right), \quad (1)$$

with the time t_{meas}, taken between 50% of the falling slope of V_{bias} and V_{SQUID} exceeding a defined trigger level V_{trig}, when the junction switches into the resistive state. x denotes to the ratio of rising to falling slope per period $T = 1/f$ of the drive signal. The routine is repeated 200 times for every magnetic field value with all critical current values being recorded.

III. INDUCTANCE EFFECTS IN SINGLE JOSEPHSON JUNCTIONS

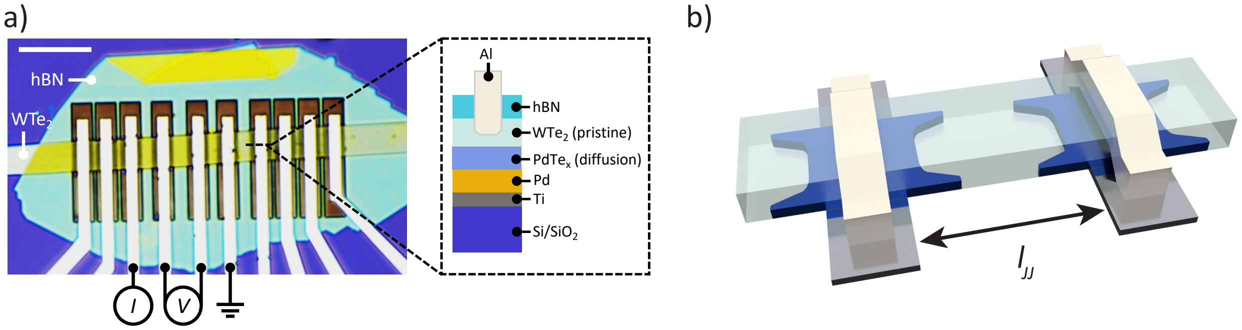

FIG. S2. a) Optical image of multiple JJs formed in a WTe_2 crystal. An illustration of the quasi four-terminal measurement scheme is illustrated at the bottom of the image. Visible is the elongated WTe_2 flake in horizontal direction, lying on vertically oriented Pd bottom contacts. The scale bar is 10 µm. The fabricated layer sequence of the heterostructure across the black dashed line is shown on the right. b) Illustration of a single Josephson junction with the effective diffusion profile of $PdTe_x$ highlighted in blue.

The complex switching behavior of the inductive SQUID can be simplified by investigating a single Josephson junction (JJ). In the following section we present the study on JJs of different lengths. The governing transport regime can be shifted from being dominated by kinetic inductance to the Josephson inductance as the junction length increases.

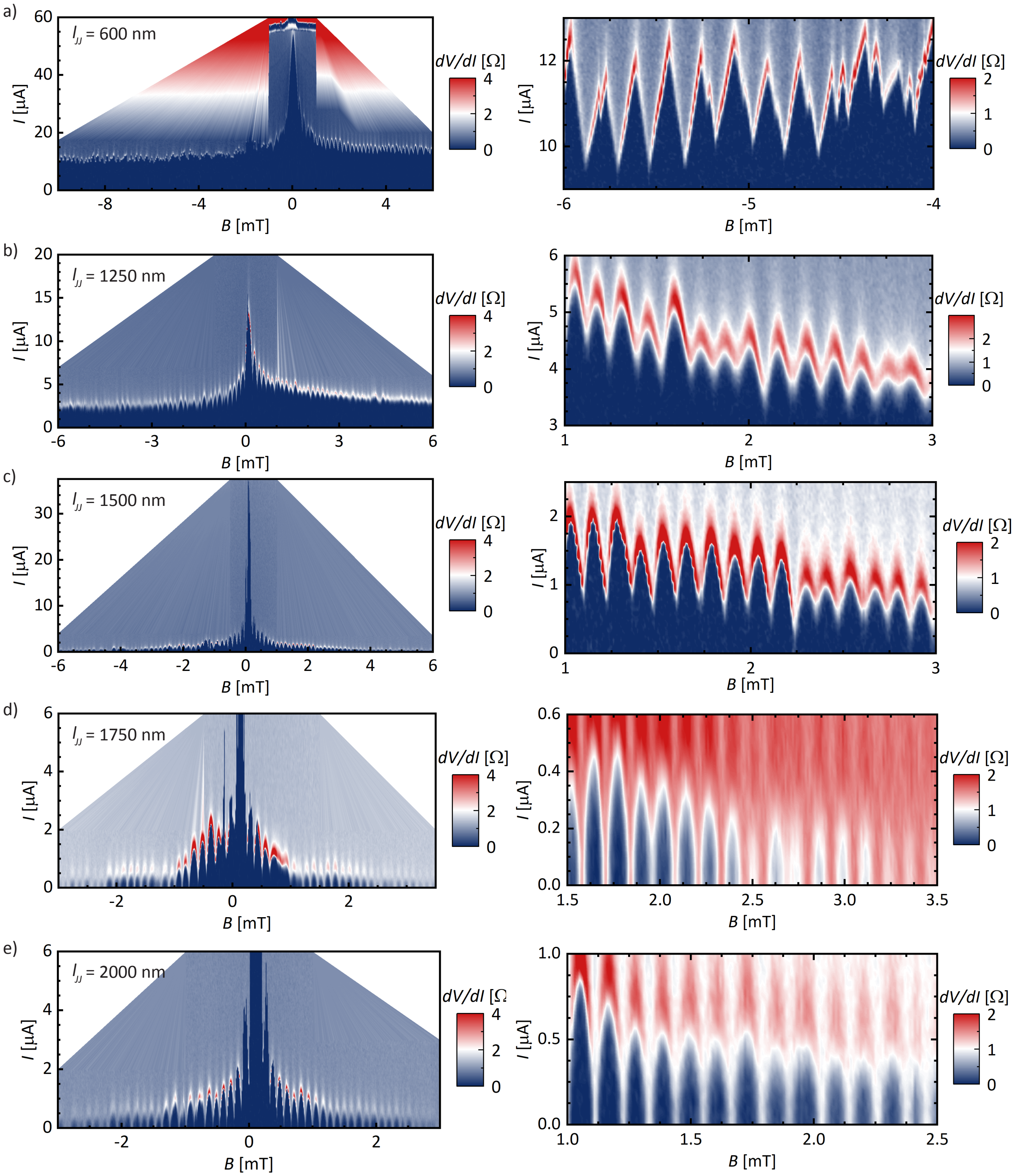

FIG. S3. Differential resistance as a function of perpendicular magnetic field and applied bias current for different junction lengths l_{JJ}. The length of the JJs increases from panels a) through e), as labelled in the top left corner of the plots on the left. The plots in the right column are a zoom in to the data to their left.

Fig. S2 a) shows an optical image of the device with its multiple JJs on an elongated WTe_2 flake. The external superconducting contacts are formed by aluminium. A cross section through the device is shown on the right. The effective diffusion profile of $PdTe_x$ inside a single junction is illustrated in Fig. S2 b).

It has recently been shown that Pd diffuses inhomogeneously into the WTe_2 crystal, with greater extent along the edges compared to the bulk [7]. The effective diffusion profile of the superconducting compound $PdTe_x$ is illustrated in blue in Fig. S2 b). The inhomogeneous diffusion profile has extensive consequences for the electronic transport. First, the enhanced diffusion-length along the edges of WTe_2 could lead to an increased role of those edges in the Josephson transport. This is beneficial in case of topological edge/hinge states, since it would diminish the contribution from trivial bulk states. Second, the diffusion channels could form a nano-inductor with high kinetic inductance $L_K \propto l/(wd)$ [10], governed by the length l, width w and thickness d of the diffusion strip.

We have fabricated a series of JJs with different spacing between the Pd bottom contacts, ranging from $l_{JJ} = 600\,\mathrm{nm}$ to $2\,\mu\mathrm{m}$. DC-current measurements are performed in four-terminal configuration, as illustrated in Fig. S2 a), with a standard lock-in technique at the base temperature of the cryostat $\sim 30\,\mathrm{mK}$. Figure S3 summarizes the differential resistance as a function of applied perpendicular magnetic field and current bias for the different junctions. Each row presents a junction length with the full range data shown on the left and a zoom-in of the data on the right. We begin with the shortest junction length $l_{JJ} = 600\,\mathrm{nm}$ in Fig. S3 a). On a macroscopic scale, the critical current envelope, at which the junction switches from zero to finite resistance, peaks sharply around zero magnetic field and decays only slowly towards higher field values. A zoom in to the data, presented in the right panel of Fig. S3 a), reveals sharp sawtooth-like oscillations that are aperiodic in magnetic field. The oscillation nodes are lifted from zero. The data resemble the characteristics of an asymmetric SQUID that is formed inside the WTe_2 junction by the spatially inhomogeneous $PdTe_x$ along the edges of the crystal. The asymmetry in critical current of the two junctions, I_c^r/I_c^w, can be quantified by the oscillation range in current amplitude between $I_c^{low} \sim 9.7\,\mu\mathrm{A}$ and $I_c^{high} \sim 12.4\,\mu\mathrm{A}$. We find $I_c^r/I_c^w = (I_c^{high} + I_c^{low})/(I_c^{high} - I_c^{low}) \sim 8.1 \gg 1$ [11], with I_c^r and I_c^w denoting the high and low critical current of the reference and weak junction, respectively.

With increasing junction length, shown in direction of the lower panels in Fig. S3, the overall shape of $I_c(B)$ resembles a "Fraunhofer" pattern of a single JJ in a magnetic field.

In more detail, with increasing junction length, the oscillation amplitude reduces and the shape of the oscillations in magnetic field becomes more sinusoidal. Furthermore, the nodal offset from zero vanishes.

In an asymmetric SQUID, such as it is the case for the shortest junction in Fig. S3 a), I_c as a function of magnetic field maps the CPR of the weak JJ with smaller critical current [12]. The sawtooth-like CPR could be interpreted as being in the ballistic long junction limit, originating from the topological edge states of the material [13]. We argue, however, that this interpretation is unlikely to be the case in the presented device when the aperiodicity and inconsistent change in amplitude of the oscillations are taken into account. Instead, the linear behavior in magnetic field is attributed to inductance effects in the junction [14–18].

The evolution of $I_c(B)$ with junction length, from a sawtooth-like to a sinusoidal interference pattern, can be explained consistently in the framework of a SQUID with flux screening that is formed by the extended $PdTe_x$ arms along the WTe_2 crystal edges. We assume that the $PdTe_x$ diffusion along the edges is similar for all junctions lengths and gives a constant contribution to the kinetic loop inductance L_K. As the junction length increases, so does the ratio between Josephson inductance L_J and kinetic inductance of the diffusion layer L_K:

$$\frac{L_J}{L_K} = \frac{\Phi_0}{2\pi L_K I_c}. \tag{2}$$

Longer junctions, due to their smaller value of I_c, have an increased Josephson inductance. If L_J exceeds the kinetic inductance that originates from the $PdTe_x$ diffusion crystal, the phase difference over the junction will be determined by the external flux ϕ_x and a sinusoidal interference pattern is observed.

IV. VERIFICATION OF THE INDUCTANCE EFFECTS

Consistently with the picture of inductance effects, the slope $dI_c/d\phi_x$, attributed to the phase change in the reference junction, yields the same inductance value for the long range data ($L_r^\diamond$) Fig. S4 a) and the SQUID oscillations ($L_r^\star$) in Fig. S4 b) and the long range data ($L_r^\diamond$) in Fig. S4 b). We find $L_r^\star \sim 81\,\mathrm{pH}$ and $L_r^\diamond \sim 166\,\mathrm{pH} \sim 2L_r^\star$. The factor of 2 arises, when the reference junction is considered a symmetric SQUID itself, due to the inhomogeneous $PdTe_x$ diffusion [7], as illustrated in Fig. S4 c). In one case, the flux threads the area of the large Nb SQUID with an effective inductance $L_r^\diamond/2$, while in the other, it threads the reference junction area. The good agreement of $L_r^\diamond \sim 2L_r^\star$ was obtained by using the center-

f_w	Sin(φ)	Sin(φ)	Linear 2π	Long bal. 4π
f_r	Sin(φ)	Dayem	Linear 2π	Long bal. 4π
I_c^w [µA]	60	10	3.8	5
L_w [pH]	450	400	200	80
I_c^r [µA]	104	154	160	160
L_r [pH]	270	-	80	80

TABLE S1. **Summary of device parameters used in the fits of Fig. S5.** f_{w} and f_{r} denote to the normalized CPR function of the weak and reference junction used in the fit model. In case of the reference junction being modelled as Dayem bridge, all inductances are incorporated through the slope $1/L = dI/d\phi_{\mathrm{x}}$ of the CPR. "Long bal. 4π" is the abbreviation for a 4π periodic topological JJ in the ballistic long junction limit.

to-center distance between the Nb contacts in the reference junction. The measure can be explained by the sample design, where the flux through half of the superconducting contact is screened into the junction [19].

V. COMPARISON OF CPR CONFIGURATIONS IN THE FITTING PROCEDURE

In the main text of the manuscript, we have fitted the data under the assumption that both junctions in the SQUID are in the short ballistic limit. The assumption was based on the non-linearity of $I_{\mathrm{c}}(\phi_{\mathrm{x}})$ in the rising slope and the fact that the phase φ_{r} of the reference junction does not remain fixed and therefore requires an abrupt jump in the CPR function.

In Fig. S5 we present additional fits to the data $I_{\mathrm{c}}\phi_{\mathrm{x}}$ of the main text, based on different CPR functions of the two junctions. Details of fit parameters are summarized in Tab. S1.

Figure S5 a) addresses both, the weak junction and the reference junction a sinusoidal CPR function. In order to obtain the instability of the reference junction phase, $I_{\mathrm{c}}^{\mathrm{w}}$ has to be heavily increased compared to the observed data range. The amplitude of the reference junction is adjusted such that $I_{\mathrm{c}}^{\mathrm{r}}$ =164 µA$-I_{\mathrm{c}}^{\mathrm{w}}$. The increased $I_{\mathrm{c}}^{\mathrm{w}}$ gives rise to additional excited SQUID states that extend below the data. The series inductances of both SQUID arms, L_{w} and L_{r} are adjusted such that the fit matches the slope of the data for a chosen $I_{\mathrm{c}}^{\mathrm{w}}$. In this case it is not possible to determine one single parameter set that fits the data.

Reason for this is, that the limited data range of the measurement is not enough to picture higher excited branches with very low switching probability.

Following a similar argument, Fig. S5 b) assumes a sinusoidal CPR for the weak junction, while treating the reference junction as Dayem bridge. The approach would hold, if $\mathrm{PdTe_x}$ had created a superconducting short in the reference junction. Here, all inductance effects are included in the slope of the CPR, according to $1/L_\mathrm{r} = dI_\mathrm{c}/d\phi_\mathrm{x}$, resulting in a periodicity that exceeds 2π. Visible around the maximum I_c is an overshoot of the fit, that originates from the maximization process of I_c. Due to the shallow slope of the Dayem bridge, φ_w continues to evolve in flux beyond the maximum value of $I_\mathrm{w} = I_\mathrm{c}^\mathrm{w} Sin(\phi_\mathrm{r} + \phi_\mathrm{x})$ until the point when $I_\mathrm{w} < I_\mathrm{r}$.

In a third option, plotted in Fig. S5 c), both CPR functions are considered to be of a linear inductor due to the dominating inductance effects.

Last, Fig. S5 d) considers the weak and reference junction to be in the topological regime. Both junctions are modelled to be in the ballistic long junction limit with its characteristic sawtooth CPR. It should be noted, that the effective fit does not change significantly, if the reference junction is considered to be in the ballistic short junction limit, as can be seen from the presented fits above. The red and blue branch of the fit correspond to the Majorana bound states with opposite parity, shifted by a phase of 2π relative to each. The extension of the fit below the fit originates form the included inductance $L_\mathrm{w} = L_\mathrm{r} = 80\,\mathrm{pH}$ in the SQUID arms.

In conclusion, none of the four alternative scenarios matches the experimental data in all its features. In particular the curvature of the rising slope is best reproduced by the both junctions being in the short ballistic limit, as presented in the main text.

VI. INSTABILITY OF THE REFERENCE JUNCTION PHASE φ_r

We argue in the following section that current crossings in a multivalued $I_\mathrm{c}(\phi_\mathrm{x})$ directly imply that the reference junction phase φ_r in an asymmetric SQUID can not remain fixed at its maximum value.

Let us assume that the initial situation is as observed in Fig. 3 a) of the main text, such that $I_\mathrm{c}(\phi_\mathrm{x})$ has self-crossings. Each crossing would be connected to two values in total flux, called $\phi_{\mathrm{tot},1}$ and $\phi_{\mathrm{tot},2}$, respectively. If φ_r remained fixed and only φ_w evolved, the device would be analogue to an ac-SQUID, containing only a single JJ. The current I circulating

in such a device is described by $I = I_c f(\phi_{tot})$, with I_c being the critical current of the JJ and f being its CPR. The total flux in the ac-SQUID with inductance L is given by $\phi_{tot} = \phi_x + I(\phi_{tot})L$. Since I and ϕ_x are identical at the crossing point, the two equations directly imply that $\phi_{tot,1} = \phi_{tot,2}$. For this reason it is not possible to obtain self-crossings in $I_c(\phi_x)$ if only one junction phase evolves.

VII. FREQUENCY DEPENDENCE OF THE SWITCHING STATISTICS

The formation of higher vorticity states in the SQUID can be understood in terms of a a particle with mass moving in a two-dimensional SQUID potential, as described by Ref. [15]. This two-dimensional potential for an inductive DC SQUID can be seen as an analogue to the standard washboard potential of a single JJ [20]. In the following discussion we introduce the nomenclature of a ground state and higher vorticity state of the SQUID to describe the data [15]. These terms denote the I_c values above and below the crossing point of the multivalued $I_c(\phi_x)$ measurement and relate to the circulating current in the system. Figure S6 a) plots the average values of the switching distributions for the ground state and the higher vorticity state in red and turquoise, respectively. The frequency of the applied ac-current is $f = 1777\,\mathrm{Hz}$. Following the same color code, Fig. S6 b) traces the number of switching events in each of the states. At each magnetic field value, the critical current is measured 200 times in total and the switching events are distributed amongst the two visible states. The mean values of the ground state and higher vorticity state are ~ 177.3 and ~ 22.7 switching events per magnetic field step, relating to a ratio $r \sim 0.128$. We observe the higher vorticity SQUID state down to the lowest applied sawtooth-frequency $f = 5\,\mathrm{Hz}$ in Fig. S7 a). In this case, the mean value of the higher-order state is reduced to ~ 19.4 switching events per magnetic field value, resulting in $r \sim 0.107$.

REFERENCES

[1] P. Blake, E. Hill, A. Castro Neto, K. Novoselov, D. Jiang, R. Yang, T. Booth, and A. Geim, Making graphene visible, Applied Physics Letters **91**, 063124 (2007).

[2] F. Ye, J. Lee, J. Hu, Z. Mao, J. Wei, and P. X.-L. Feng, Environmental instability and degradation of single-and few-layer WTe_2 nanosheets in ambient conditions, Small **12**, 5802 (2016).

[3] F. Hou, D. Zhang, P. Sharma, S. Singh, T. Wu, and J. Seidel, Oxidation kinetics of WTe_2 surfaces in different environments, ACS Applied Electronic Materials **2**, 2196 (2020).

[4] H. Liu, N. Han, and J. Zhao, Atomistic insight into the oxidation of monolayer transition metal dichalcogenides: from structures to electronic properties, Rsc Advances **5**, 17572 (2015).

[5] Y. Zhao, H. Liu, J. Yan, W. An, J. Liu, X. Zhang, H. Wang, Y. Liu, H. Jiang, Q. Li, *et al.*, Anisotropic magnetotransport and exotic longitudinal linear magnetoresistance in WTe_2 crystals, Physical Review B **92**, 041104 (2015).

[6] P. Zomer, M. Guimarães, J. Brant, N. Tombros, and B. Van Wees, Fast pick up technique for high quality heterostructures of bilayer graphene and hexagonal boron nitride, Applied Physics Letters **105**, 013101 (2014).

[7] M. Endres, A. Kononov, M. Stiefel, M. Wyss, H. S. Arachchige, J. Yan, D. Mandrus, K. Watanabe, T. Taniguchi, and C. Schönenberger, Transparent Josephson junctions in higher-order topological insulator WTe_2 via Pd diffusion, Phys. Rev. Materials **6**, L081201 (2022).

[8] A. Kononov, M. Endres, G. Abulizi, K. Qu, J. Yan, D. G. Mandrus, K. Watanabe, T. Taniguchi, and C. Schönenberger, Superconductivity in type-II Weyl-semimetal WTe_2 induced by a normal metal contact, Journal of Applied Physics **129**, 113903 (2021).

[9] D. I. Indolese, P. Karnatak, A. Kononov, R. Delagrange, R. Haller, L. Wang, P. Makk, K. Watanabe, T. Taniguchi, and C. Schönenberger, Compact SQUID realized in a double-layer graphene heterostructure, Nano Letters **20**, 7129 (2020).

[10] A. J. Annunziata, D. F. Santavicca, L. Frunzio, G. Catelani, M. J. Rooks, A. Frydman, and D. E. Prober, Tunable superconducting nanoinductors, Nanotechnology **21**, 445202 (2010).

[11] A. Kononov, G. Abulizi, K. Qu, J. Yan, D. Mandrus, K. Watanabe, T. Taniguchi, and C. Schönenberger, One-dimensional edge transport in few-layer WTe_2, Nano letters **20**, 4228 (2020).

[12] M. L. Della Rocca, M. Chauvin, B. Huard, H. Pothier, D. Esteve, and C. Urbina, Measurement of the Current-Phase Relation of Superconducting Atomic Contacts, Physical Review Letters **99**, 127005 (2007).

[13] A. Murani, A. Kasumov, S. Sengupta, Y. A. Kasumov, V. Volkov, I. Khodos, F. Brisset, R. Delagrange, A. Chepelianskii, R. Deblock, H. Bouchiat, and S. Guéron, Ballistic edge states in Bismuth nanowires revealed by SQUID interferometry, Nature Communications **8**, 1 (2017).

[14] A. Murphy and A. Bezryadin, Asymmetric nanowire SQUID: Linear current-phase relation, stochastic switching and symmetries, Physical Review B **96**, 094507 (2017).

[15] V. Lefevre-Seguin, E. Turlot, C. Urbina, D. Esteve, and M. H. Devoret, Thermal activation of a hysteretic dc superconducting quantum interference device from its different zero-voltage states, Physical Review B **46**, 5507 (1992).

[16] F. Friedrich, P. Winkel, K. Borisov, H. Seeger, C. Sürgers, I. M. Pop, and W. Wernsdorfer, Onset of phase diffusion in high kinetic inductance granular aluminum micro-squids, Superconductor Science and Technology **32**, 125008 (2019).

[17] D. Hazra, J. R. Kirtley, and K. Hasselbach, Nano-superconducting quantum interference devices with suspended junctions, Applied Physics Letters **104**, 152603 (2014).

[18] H. Dausy, L. Nulens, B. Raes, M. J. Van Bael, and J. Van de Vondel, Impact of Kinetic Inductance on the Critical-Current Oscillations of Nanobridge SQUIDs, Physical Review Applied **16**, 024013 (2021).

[19] S. Ghatak, O. Breunig, F. Yang, Z. Wang, A. A. Taskin, and Y. Ando, Anomalous Fraunhofer patterns in gated Josephson junctions based on the bulk-insulating topological insulator $BiSbTeSe_2$, Nano Letters **18**, 5124 (2018).

[20] M. Tinkham, *Introduction to Superconductivity*, 2nd ed. (Dover Publications, 2004).

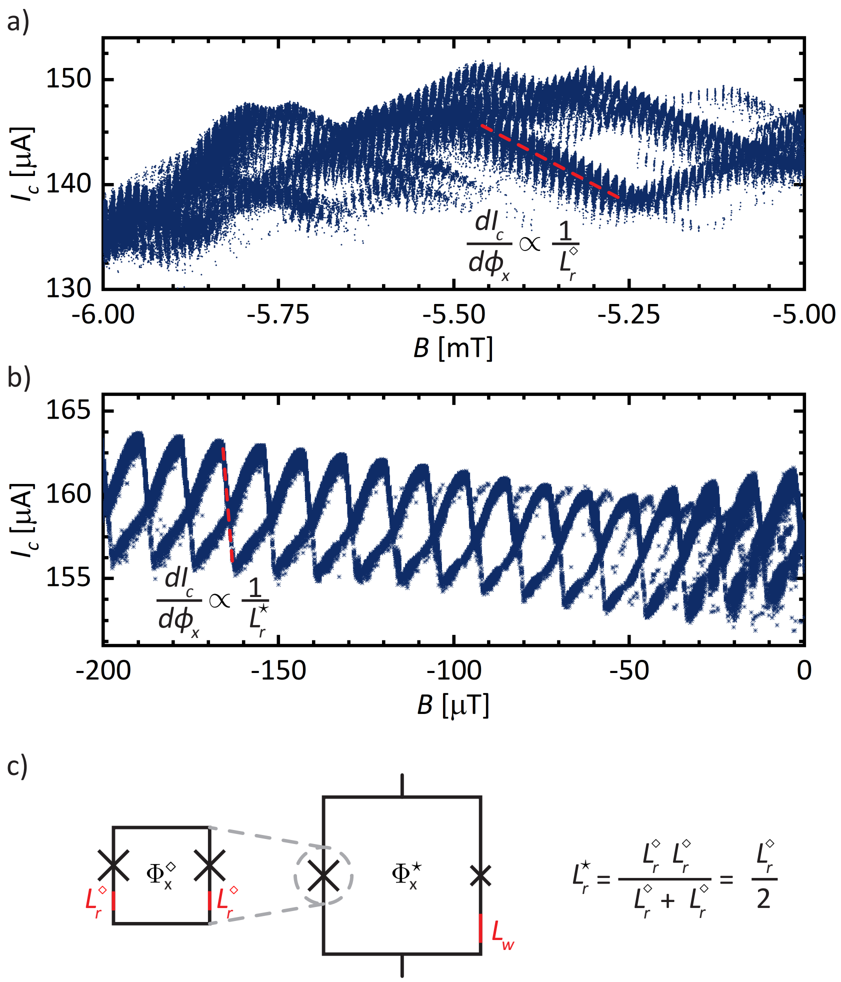

FIG. S4. **Extraction of the reference junction series inductance L_r for different magnetic field ranges through the linear slope of the oscillations.** a) Long range data of the SQUID that purely resembles the behavior of the reference junction. The SQUID oscillations are superimposed on top of the long range oscillations. b) High resolution measurement of the SQUID oscillations at a point in magnetic field. c) Schematic illustration to explain the factor two difference between the extracted slopes $L_\mathrm{r}^\diamond$ and $L_\mathrm{r}^\star$.

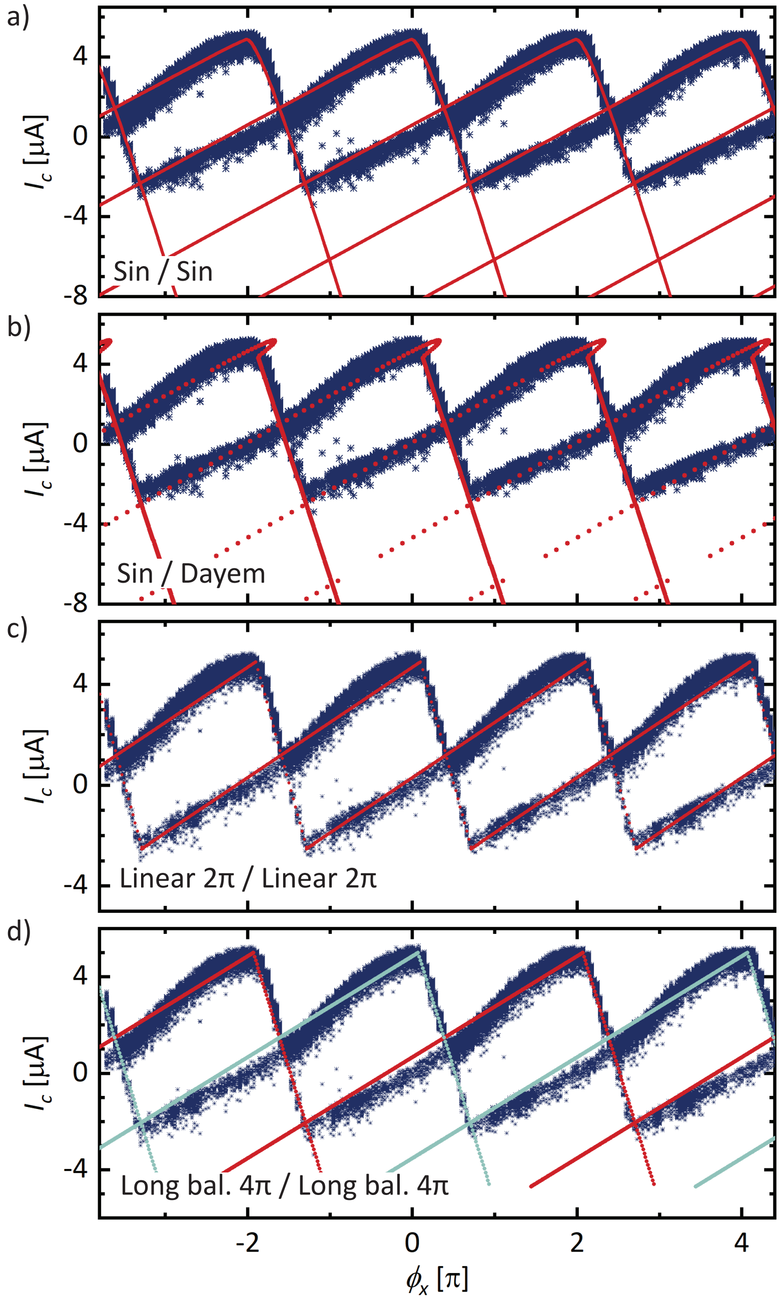

FIG. S5. **Comparison of fit models with different CPR functions.** The weak junction and the reference junction, respectively, are assumed to have a) both a sinusoidal CPR, b) a sinusoidal CPR and the behavior of Dayem bridge and c) both a linear CPR of an inductor. d) Topological junction in the ballistic long junction limit with a 4π-periodic CPR. The red and green branch correspond to two parity states that are shifted by a phase of 2π relative to each through quantum phase slips.

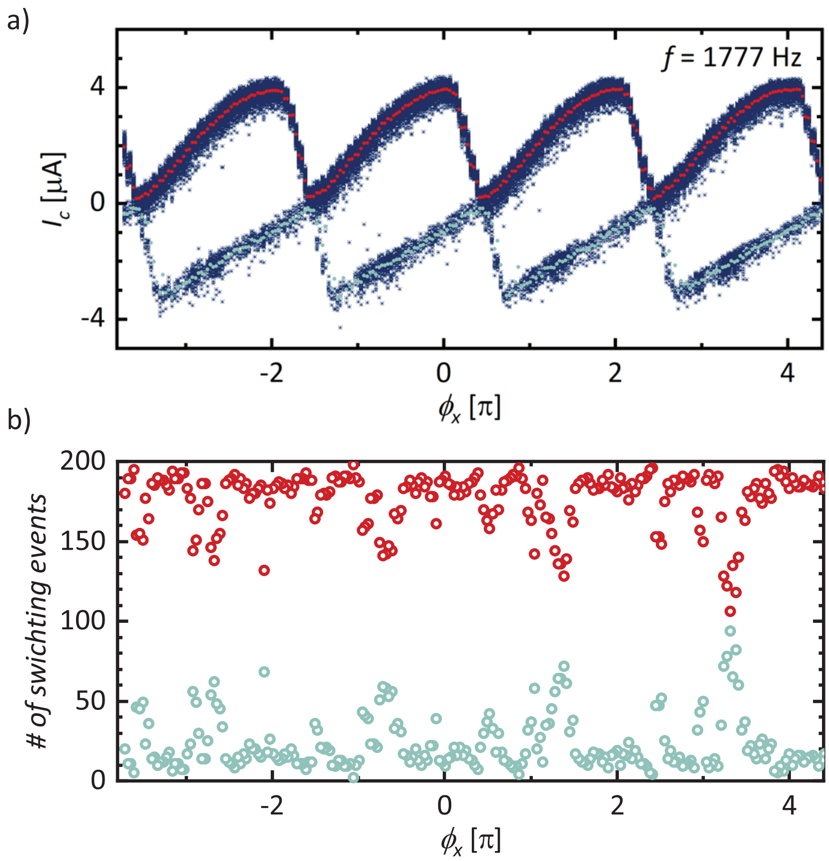

FIG. S6. **Occupation of the zeroth and higher vorticity states at applied ac frequency** $f = 1777\,\mathrm{Hz}$**.** a) SQUID oscillations with color-coded average value for the zeroth and higher vorticity state above and below the current crossing point. b) Number of switching events for the two intertwined branches. Each magnetic field step contains 200 switching events, of which $\sim 13\%$ are occupying the higher vorticity state below the crossing point of I_c values.

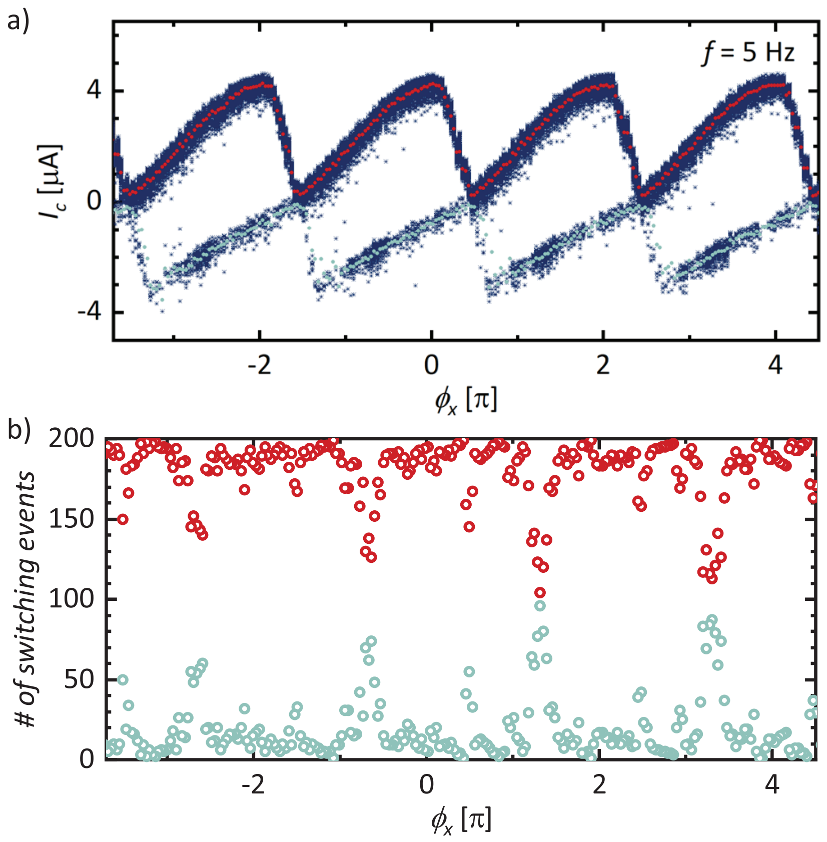

FIG. S7. **Occupation of the zeroth and higher vorticity SQUID states at applied ac frequency** $f = 5\,\mathrm{Hz}$**.** a) SQUID oscillations with color-coded average value for the zeroth and higher-order state above and below the current crossing point. b) Number of switching events for the two intertwined branches. Each magnetic field step contains 200 switching events, of which $\sim 11\%$ are occupying the higher vorticity state below the crossing point of I_c values.
